# The hemodynamic effects of bolus norepinephrine and phenylephrine in the treatment of hypotensive events in preeclamptic women with severe features following spinal anesthesia during cesarean section: a randomized controlled trial

**DOI:** 10.3389/fphar.2026.1783419

**Published:** 2026-06-25

**Authors:** Lili Zou, Hongping Li, Yongqiang Shi, Nan Xi, Yi Chen

**Affiliations:** 1 Department of Anesthesiology and Perioperative Medicine, General Hospital of Ningxia Medical University, Yinchuan, Ningxia, China; 2 Department of Anesthesiology, Peking University First Hospital Ningxia Women and Children’s Hospital (Ningxia Hui Autonomous Region Maternal and Child Health Hospital), Yinchuan, Ningxia, China

**Keywords:** cesarean section, hemodynamic effects, hypotension, norepinephrine, phenylephrine, preeclampsia with severe features, spinal anesthesia

## Abstract

**Study Objective:**

To investigate the hemodynamic effects of rescue bolus administration of norepinephrine (NE) and phenylephrine (PE) in the treatment of hypotensive events in preeclamptic women with severe features following spinal anesthesia during cesarean section.

**Methods:**

A total of 40 preeclamptic women with severe features (*n* = 20 per group) experienced hypotensive events (defined as SBP below 80% of baseline value) following spinal anesthesia were randomly receive 6 µg NE or 75 µg PE as a rescue measure. The primary outcomes were the maximal changes and the percentage of these changes in hemodynamic indicators, including systolic blood pressure (SBP), heart rate (HR), cardiac output (CO), and systemic vascular resistance (SVR) within a 3-min period following the administration of NE or PE. Additionally, the occurrence of maternal bradycardia, reactive hypertension, analysis of umbilical artery blood gas, and assessment of neonatal Apgar score were recorded.

**Results:**

In the NE group, there was a lower magnitude of maximal changes (85.40 ± 17.76 vs. 71.10 ± 11.88 beats/min, *p* = 0.005; 7.78 ± 1.98 vs. 6.11 ± 1.44 L/min, *p* = 0.004) after vasopressor administration, accompanied by lower respective percentages of change (−15.54% ± 11.49% vs. −23.41% ± 10.03%, *p* = 0.026; −1.75% ± 6.50% vs. −16.56% ± 10.21%, *p* < 0.001) for HR and CO, as well as a decreased incidence of bradycardia (5.0% vs. 30.0%, *p* = 0.030) compared to the PE group.

**Conclusion:**

Either 6 µg NE or 75 µg PE can effectively rescue and exhibit comparable efficacy in managing hypotensive events following spinal anesthesia in preeclamptic women with severe features. However, NE demonstrates superior capability in maintaining CO and HR.

## 1 Introduction

The management of the perioperative period in preeclamptic women, which affects approximately 3%–8% of pregnancies, presents a challenge due to the presence of hypertension, systemic edema, decreased blood volume, and ventricular diastolic dysfunction (Siddiqui et al., 2019; Hofmeyr et al., 2017). Spinal anesthesia is a widely employed method for cesarean section, offering optimal analgesia, a simplified procedure, high reliability, and mitigating additional postoperative pain, sedation, postpartum hemorrhage, and associated complications (Lim et al., 2018). Additionally, spinal anesthesia offers specific benefits for preeclamptic women by effectively mitigating the risks associated with glottis edema-induced intubation failure and excessive blood pressure-induced hemorrhagic stroke during intubation. Furthermore, it can reduce resistance in the uterine artery, thereby enhancing intervillous blood flow and optimizing placental gas exchange. Therefore, spinal anesthesia remains a highly advantageous option (Siddiqui et al., 2019; Russell, 2020).

The blood pressure of preeclamptic women was significantly elevated compared to that of normotensive women due to systemic arteriolar constriction and increased systemic vascular resistance (SVR), thereby reducing the likelihood of hypotensive events following spinal anesthesia (Siddiqui et al., 2019; Nikooseresht et al., 2016). The occurrence of sudden or severe hypotensive events, however, can further compromise uterine placental perfusion and subsequently impact maternal and infant outcomes (Basaran et al., 2015). Vasopressors have traditionally been regarded as a primary strategy for managing hypotensive events following spinal anesthesia in preeclamptic women. However, the heightened sympathetic nerve excitability observed in such women often increases their sensitivity to vasopressors. Even smaller doses of vasopressors can effectively address hypotensive events induced by spinal anesthesia (Henke et al., 2013). Maternal cardiac output (CO) plays a crucial role in supplying blood to the uterus and placenta. Therefore, monitoring hemodynamic indicators such as CO and SVR can offer valuable guidance for anesthesia management decisions, including fluid administration, diuretic usage, vasodilator administration, as well as assessing the effectiveness of vasopressors in treating hypotensive events (Dyer et al., 2011).

The current evidence suggests that norepinephrine (NE) exhibits comparable efficacy to phenylephrine (PE) in the management of maternal hypotensive events in normotensive women following spinal anesthesia, while demonstrating minimal impact on heart rate (HR) and CO (Belin et al., 2023). Simultaneously, appropriate fluid management was administered to optimize maternal uterine placental perfusion and improve maternal and neonatal outcomes. However, limited evidence exists regarding the utilization of NE in preeclamptic women, particularly concerning to the monitoring of hemodynamic indicators. The objective of this study was to investigate the hemodynamic effects of rescue bolus administration of NE and PE in the treatment of hypotensive events in preeclamptic women with severe features following spinal anesthesia during cesarean section.

## 2 Materials and methods

### 2.1 Study design and setting

The present study was a prospective, double-blind, randomized controlled trial that received approval from the Ethics Committees of General Hospital of Ningxia Medical University (No. KYLL-2021-918). The trial was registered on ClinicalTrials.gov (NCT05035485) and conducted from September 2022 to January 2024 with the informed consent of all participating preeclamptic women who provided signed consent prior to inclusion.

The inclusion criteria were as follows: singleton pregnancy of ≥32 gestational weeks, aged between 18 and 45 years, with a diagnosis of preeclampsia undergoing cesarean section under spinal anesthesia; ASA physical status II-III. The exclusion criteria were as follows: baseline systolic blood pressure (SBP) ≥180 mmHg; Body mass index ≥40 kg/m^2^; contraindication to spinal anesthesia; presence of eclampsia or chronic hypertension; severe complications related to hypertension or preeclampsia, including those affecting the nervous and cardiovascular system, pulmonary edema, or left ventricular dysfunction; fetal distress or known abnormalities of fetal development. The diagnostic criteria for preeclampsia with severe features were as follows (Russell, 2019): if SBP is ≥ 160 mmHg or DBP is ≥ 110 mmHg on two occasions with at least a 4-h interval, and if there is new onset of one or more of the following coexisting conditions: thrombocytopenia (<100,000 platelets 10^9^/L), renal insufficiency (serum creatinine concentration exceeding 1.1 mg/dL or a doubling in value in the absence of other renal diseases), impaired liver function (the blood concentration of liver transaminases is twice the normal level), pulmonary edema, or cerebral or visual symptoms.

### 2.2 Monitoring

The preeclamptic women with severe features underwent continuous monitoring of electrocardiogram (ECG), noninvasive blood pressure, and percutaneous pulse oxygen saturation (SpO_2_). Radial artery catheters were inserted under local anesthesia and guided by a portable ultrasonic diagnostic scanner, while hemodynamic monitoring equipment (FloTrac/Vigileo, EV1000, Edwards, USA) was connected. Subsequent SBP, HR, CO, stroke volume (SV), stroke volume variation (SVV), and cardiac index (CI) were continuously monitored for all women. Central venous pressure (CVP) was not measured. In accordance with the report by Dyer et al. (2018a) we also set the CVP at 5 mmHg for the subsequent determination of SVR. All women received an 18G intravenous catheter in the upper limb for crystalloid infusion and intravenous rescue bolus administration of NE and PE. No fluid preload was administered prior to spinal anesthesia, while maintaining crystalloid infusion at a rate of approximately 1 mL/min. The mean values of each hemodynamic indicator were monitored five times (updated every 20 s; with a difference of less than 10%) in a supine position with a left-leaning operating table at 15° in a resting state serving as the baseline value before spinal anesthesia. Subsequently, these indicators were continuously recorded (updated every 20 s) until the completion of surgery.

### 2.3 Anesthesia and intervention

After puncturing the L2-3 vertebral interspace with a 25G spinal needle in the left lateral position, an intrathecal injection of 12.5 mg bupivacaine (0.5%; w/v) was administered upon identification of cerebrospinal fluid. Following spinal anesthesia, the uterus was tilted at a 15° angle to the left while crystalloid fluid was infused at a rate of 5–6 mL/kg/h. The block height must exceed T6 within 10 min following spinal anesthesia (as determined by cold stimulation or a sterile needle) prior to granting permission for the surgical procedure. The decision to administer further vasopressor intervention was based on the occurrence of hypotensive events within 10 min following spinal anesthesia. In the absence of any observed hypotensive events (defined as SBP below 80% of baseline value), the woman would be excluded from the study. However, in cases where hypotensive events occurred, the sealed envelope would be opened, revealing a randomly generated number sequence corresponding to either the NE group or the PE group. Subsequently, depending on the outcome of opening the envelope, either 6 µg NE or 75 µg PE would be administered as a rescue measure. If SBP did not recover to ≥80% of baseline value, repeated administration would be performed.

### 2.4 Study outcomes

The primary outcomes were the maximal changes in hemodynamic indicators, including CO, SBP, HR, and SVR within a 3-min period following the administration of NE or PE. Additionally, the percentage of maximal changes after vasopressor application was also evaluated. Secondary outcomes included the maximal changes in hemodynamic indicators and the percentage of these changes relative to baseline values following spinal anesthesia, as well as the incidence of maternal bradycardia (HR < 60 beats/min; treated with 0.5 mg atropine), maternal reactive hypertension (SBP > 120% of baseline or >180 mmHg), nausea and vomiting. Other outcomes included umbilical artery blood gas analysis, neonatal Apgar score (at 1 and 5 min), and rate of transfer to the neonatal intensive care unit (NICU).

### 2.5 Statistical analyses

In our pilot study, the percentage of maximal CO changes within 3 min after PE rescue bolus (a total of 5 cases) was −15.18% ± 10.59%. We hypothesized a relative reduction of absolute 12% in the corresponding maximal changes following NE rescue bolus. A sample size of 18 women per group was required to detect this difference at the significance level of 0.05 with a power of 90%, using Two-Sample T-Tests Allowing Equal Variance (PASS15.0; NCSS). The total enrollment consisted of 40 women, with 20 women allocated to each group, in order to account for potential dropouts.

The normality of continuous data was assessed by conducting the Kolmogorov-Smirnov test. For groups conforming to a normal distribution, the comparison was conducted using an independent sample *t*-test and reported as mean ± standard deviation (SD). For groups not conforming to a normal distribution, the comparison was conducted using the Mann-Whitney U test and reported as interquartile range (IQR). The categorical data was compared using the chi-square test and reported as percentages. The repeated measures of SBP, HR, CO, and SVR were compared using ANOVA. The data were analyzed using SPSS Statistics version 23.0 (IBM SPSS, Inc., Chicago, IL). A statistical difference was considered significant at a significance level of *p* < 0.05.

## 3 Results

**FIGURE 1:**
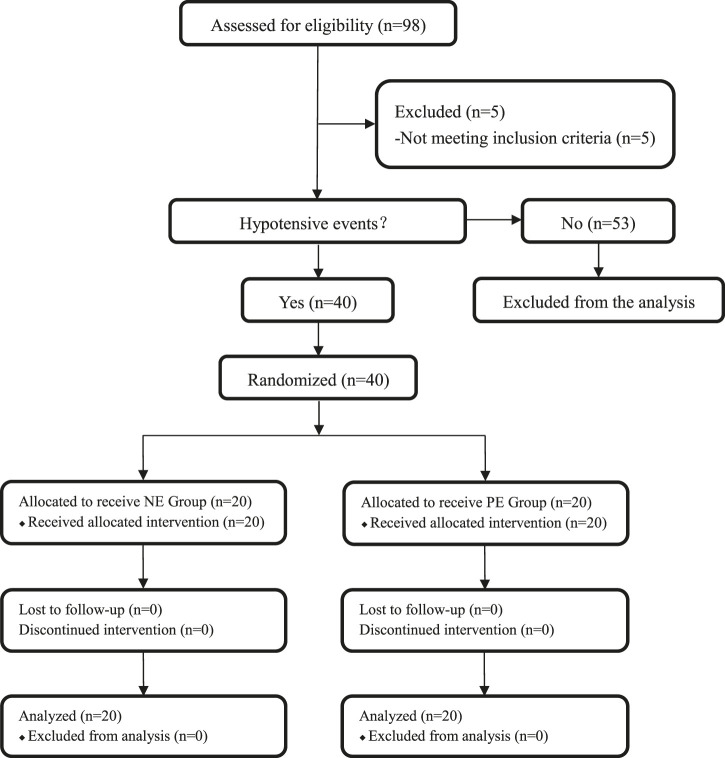
CONSORT flow chart.

The flow chart depicting the inclusion of preeclamptic women with severe features is presented in Figure 1. A total of 93 preeclamptic women with severe features who met the criteria were included, while 53 women who did not experience hypotensive events were excluded. Ultimately, a total of 40 women (20 in each group) were analyzed. The demographic characteristics of both groups exhibited similarity, as illustrated in Table 1.

**TABLE 1:** Demographic characteristics.

	NE Group (n = 20)	PE Group (n = 20)	*P* value
Age (years)	32.45 ± 4.22	32.30 ± 6.27	0.930
Body mass index (kg/m^2^)	30.45 ± 3.24	29.99 ± 3.67	0.673
Gestational age (weeks)	34 [33 - 37]	34 [32 - 36]	0.536
Block height	T5 [T4 - T6]	T5 [T4 - T6]	0.786
Time from anesthesia to delivery (min)	15.35 ± 2.52	14.45 ± 3.41	0.348
Time from skin incision to delivery (min)	3.10 ± 1.07	2.75 ± 1.37	0.374

Data were presented as mean ± SD (standard deviation) and median [quartiles].

**FIGURE 2:**
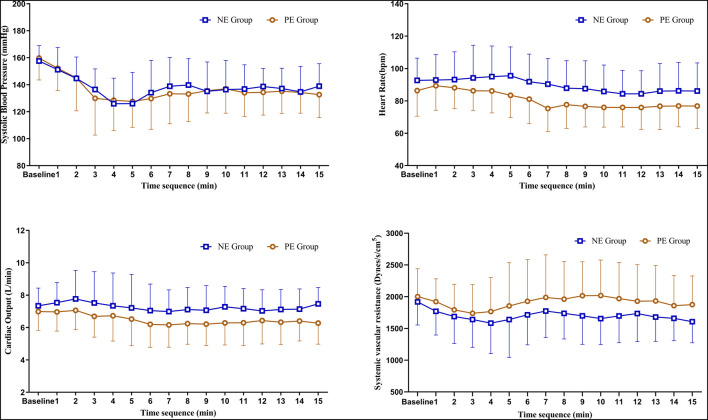
The observed trends of systolic blood pressure (SBP), heart rate (HR), cardiac output (CO), and systemic vascular resistance (SVR) in both groups within 15 min following spinal anesthesia. Data are represented as means ± standard deviation.

The hemodynamic indicators, including SBP, HR, CO, and SVR, were depicted in Figure 2 to illustrate the observed trends in both groups within 15 min following spinal anesthesia. Repeated measures ANOVA revealed a significant main effect of time on SBP (*p* < 0.001), HR (*p* < 0.001), CO (*p* = 0.007), and SVR (*p* = 0.002), along with a significant main effect of group on HR (*p* = 0.038) and CO (*p* = 0.049). In contrast, neither the main effect of group on SBP (*p* = 0.696) and SVR (*p* = 0.130) nor the intervention * group interactions (*p* = 0.607; *p* = 0.297; *p* = 0.670; *p* = 0.402) regarding SBP, HR, CO, and SVR was statistically significant.

The baseline values, the maximal changes after spinal anesthesia, and the corresponding percentage changes compared to the baseline values are presented in Table 2. Additionally, Table 2 also displays the maximal changes after vasopressor application according to different groups and their respective percentages of change. The SBP and SVR values were comparable between the two groups. The baseline values, maximal changes following spinal anesthesia, and corresponding percentage changes relative to baseline for HR and CO were comparable in different groups. However, in the NE group, there was a lower magnitude of maximal changes (85.40 ± 17.76 vs. 71.10 ± 11.88 beats/min, *p* = 0.005; 7.78 ± 1.98 vs. 6.11 ± 1.44 L/min, *p* = 0.004) after vasopressor administration, accompanied by lower respective percentages of change (−15.54% ± 11.49% vs. −23.41% ± 10.03%, *p* = 0.026; −1.75% ± 6.50% vs. −16.56% ± 10.21%, *p* < 0.001) compared to the PE group.

**TABLE 2:** Changes in hemodynamic variables.

	NE Group (n= 20)	PE Group (n= 20)	*P* value
Systolic blood pressure (mmHg)			
Baseline	157.6 ± 11.39	159.80 ± 16.24	0.623
Maximal changes after spinal anesthesia	114.85 ± 9.54	113.80 ± 19.62	0.831
% of maximal changes after spinal anesthesia	-26.96 ± 5.83	-28.92 ± 8.88	0.413
Maximal changes after vasopressor application	145.25 ± 19.22	140.40 ± 18.96	0.427
% of maximal changes after vasopressor application	26.49 ± 12.61	25.17 ± 16.75	0.786
Heart rate (beats/min)			
Baseline	92.65 ± 13.79	86.35 ± 15.80	0.187
Maximal changes after spinal anesthesia	101.20 ± 16.85	93.60 ± 14.72	0.137
% of maximal changes after spinal anesthesia	9.63 ± 12.44	9.76 ± 15.99	0.977
Maximal changes after vasopressor application	85.40 ± 17.76	71.10 ± 11.88	0.005
% of maximal changes after vasopressor application	-15.54 ± 11.49	-23.41 ± 10.03	0.026
Cardiac output (L/min)			
Baseline	7.34 ± 1.10	6.99 ± 1.15	0.324
Maximal changes after spinal anesthesia	7.94 ± 2.06	7.30 ± 1.32	0.250
% of maximal changes after spinal anesthesia	8.10 ± 21.00	4.56 ± 10.05	0.501
Maximal changes after vasopressor application	7.78 ± 1.98	6.11 ± 1.44	0.004
% of maximal changes after vasopressor application	-1.75 ± 6.50	-16.56 ± 10.21	< 0.001
Systemic vascular resistance (Dynes/s/cm5)			
Baseline	1917.35 ± 363.98	2001.00 ± 440.02	0.516
Maximal changes after spinal anesthesia	1384.25 ± 394.92	1509.00 ± 466.22	0.367
% of maximal changes after spinal anesthesia	-28.38 ± 13.44	-25.09 ± 15.78	0.482
Maximal changes after vasopressor application	1886.00 ± 480.94	2126.60 ± 632.19	0.184
% of maximal changes after vasopressor application	44.89 ± 58.96	43.01 ± 18.74	0.893

Data were presented as mean ± SD (standard deviation).

The incidence of bradycardia was significantly higher in the PE group compared to the NE group (30.0% vs. 5.0%, *p* = 0.030). Furthermore, there were no significant differences observed in maternal and neonatal outcomes between the two groups, as presented in Table 3.

**TABLE 3:** Maternal and neonatal outcomes.

	NE Group (n= 20)	PE Group (n= 20)	*P* value
Bradycardia, n (%)	1 (5.0)	6 (30.0)	0.030
Hypertension, n (%)	2 (10.0)	1 (5.0)	0.545
Nausea andvomiting, n (%)	7 (35.0)	6 (30.0)	0.736
pH of umbilical artery	7.31 ± 0.04	7.31 ± 0.03	0.992
P_CO2_ of umbilical artery (mmHg)	46.44 ± 5.17	45.86 ± 4.73	0.713
P_O2_ of umbilical artery (mmHg)	19.18 ± 5.09	18.79 ± 5.22	0.813
Base excess of umbilical artery(mmol/L)	-3.13 ± 1.39	-3.40 ± 1.82	0.616
Apgar score (1 min)	8 [8 - 9]	8 [8 - 8]	0.332
Apgar score < 7 (1 min), n (%)	2 (10.0)	1 (5.0)	0.545
Apgar score (5 min)	9 [9 - 10]	9 [9 -9]	0.173
Apgar score < 7 (5 min), n (%)	0 (0.0)	0 (0.0)	> 0.999
Neonatal intensive care unit admission, n (%)	13 (65.0)	17 (85.0)	0.140

Data are presented as n (%), median [quartiles], and mean ± SD (standard deviation).

## 4 Discussion

The findings of this study demonstrated that both a 6 µg NE bolus and a 75 µg PE bolus effectively rescue hypotensive events following spinal anesthesia in preeclamptic women with severe features. In comparison to PE, NE exhibited a lesser impact on maternal maximal changes and the percentage of these changes in HR and CO.

Preeclampsia is a uterine placental disorder characterized by hypertension and proteinuria, resulting from abnormal vascular remodeling of maternal myometrial spiral arteries. This leads to heightened sensitivity to vasomotor responses, particularly prevalent in cases of early onset or preeclampsia with severe features. Consequently, it causes uterine hypoperfusion, fetal growth restriction, increased cesarean section rates, and other associated risks that contribute to elevated maternal and neonatal morbidity and mortality (Hofmeyr et al., 2017; Henke et al., 2013; US Preventive Services Task Force et al., 2017). Additionally, the cardiovascular manifestations of preeclampsia involve a reduction in plasma volume accompanied by systemic vasoconstriction. In untreated preeclamptic women with severe features, hemodynamic characteristics include elevated SVR, diminished CI, and impaired left ventricular diastolic function (Russell, 2020; Pretorius et al., 2018; Nugroho et al., 2019). The risk of surgery and anesthesia management in this particular group of women is higher compared to that of normotensive women. Currently, spinal anesthesia remains a commonly employed method. In this study, a significant decrease in blood pressure was observed among all women following spinal anesthesia, accompanied by a slight increase in CO and a notable reduction in SVR. The findings are consistent with the physiological mechanism of spinal anesthesia, which induces extensive dilation of maternal peripheral arteries and reduces SVR. Additionally, it compensates for CO by increasing HR to ensure adequate uterine placental perfusion (Dyer et al., 2008).

In this study, we primarily observed the alterations in maternal CO subsequent to the administration of vasopressors. Preeclamptic women with severe features receiving PE exhibited reductions in HR and CO, which is consistent with clinical observations and findings from previous study (Dyer et al., 2018a). After the administration of a PE rescue bolus, there can be a maximal reduction in CO of up to 30%, primarily attributed to the absence of additional β receptor activation which leads to a decrease in maternal HR. Throughout the monitoring period, both PE and NE groups exhibited stable maternal SV, showing a maximal change of approximately 10%. After the application of PE rescue bolus, there are still some women whose CO remains above the preoperative baseline value. This can be attributed to the compensatory increase in CO following spinal anesthesia, which acts as a counterbalance for the dose-dependent reduction in HR and CO caused by PE administration. The invasive and expensive nature of CO monitoring has restricted its use in general obstetric anesthesia management. Given that blood pressure and HR monitoring merely act as alternatives to CO monitoring, assessing CO can provide a more precise evaluation of maternal and fetal oxygenation levels as well as hemodynamic status (Orbach-Zinger et al., 2019; Langesæter and Dyer, 2011). The future widespread implementation of non-invasive hemodynamic monitoring may offer enhanced assurance for anesthesia decision-making, administration of vasopressors, and fluid management.

The study conducted by Dyer et al. (2018a) demonstrated that the administration of either 7.5 mg ephedrine or 50 µg PE effectively corrected hypotensive events in preeclamptic women with severe features following spinal anesthesia. Interestingly, it was observed that PE induced a significant reduction in CO and HR, with the maximal changes recorded as −12.0% ± 7.3% and −9.1% ± 3.4%, respectively. In this study, the maximal changes in CO and HR were observed to be −16.56% ± 10.21% and −23.41% ± 10.03%, respectively, which could potentially be attributed to the administration of higher doses of rescue PE, inducing a dose-dependent reduction effect on HR and CO. Wang et al. (2019) and Mohta et al. (2021) conducted studies investigating the efficacy of NE and PE boluses in managing hypotensive events in preeclamptic women following spinal anesthesia. The results demonstrated that both 4 µg NE and 50 µg PE effectively corrected maternal hypotensive events; however, the former study demonstrated a higher incidence of PE-induced bradycardia which exhibited more pronounced temporal changes, while the latter study did not yield similar findings (although the HR values were lower in the PE group during the first 5 min subsequent to spinal anesthesia). Regrettably, neither investigation delved into the hemodynamic alterations in further detail. This study further corroborates the discovery of an elevated prevalence of bradycardia in the PE group. Subsequent studies conducted by Wang et al. (2020) demonstrated that the administration of 8 µg NE and 100 µg PE effectively corrected hypotensive events and exhibited comparable efficacy in normotensive women following spinal anesthesia, with NE better maintaining HR and preserving CO. Additionally, there were no significant differences observed in neonatal outcomes between the use of rescue NE and PE.

The previous concerns have also indicated that refraining from administering higher doses of vasopressors and excessive volume overload in response to hypotensive events following spinal anesthesia may increase the risk of hemorrhagic stroke, reduce fetal oxygen supply due to increased constriction of the uterine artery, and exacerbate left ventricular dysfunction, pulmonary edema, and systemic edema in preeclamptic women with severe features (Nikooseresht et al., 2016; Basaran et al., 2015; Heesen et al., 2019). The study conducted by Ngan Kee (2017) demonstrated that the efficacy of 8 µg NE was comparable to that of 100 µg PE, with an efficacy ratio of 12.5:1, which we also adopted. However, in order to address concerns regarding reactive hypertension, we opted for a reduced rescue dosage of 75 µg PE instead of the usual 100 µg dose. Additionally, we administered a 6 µg bolus of NE to manage maternal hypotensive events with an efficacy ratio of 12.5:1. Following administration of the aforementioned rescue bolus, both NE and PE groups exhibited restoration of blood pressure to appropriate levels (145.25 ± 19.22 vs. 140.40 ± 18.96 mmHg). However, it is noteworthy that some women still experienced a significant increase in blood pressure after the application of NE and PE boluses, potentially increasing the risk of adverse events such as hemorrhagic stroke. Further high-quality evidence may be necessary to determine the optimal dosage of rescue PE and NE bolus for preeclamptic women with severe features.

Dyer et al. (2018b) utilized ephedrine and PE bolus to address hypotensive events in preeclamptic women undergoing caesarean section due to concerning fetal heart monitoring. The findings indicated that there was no significant difference in umbilical arterial pH values following the administration of either drug (7.25 ± 0.08 vs. 7.22 ± 0.10, *p* = 0.22). Similarly, Higgins et al. (2018) did not observe a significant difference in neonatal pH values when prophylactic ephedrine and PE were administered in cases of preeclampsia (7.20 ± 0.10 vs. 7.22 ± 0.07, *p* = 0.38). However, Wang et al. (2019) discovered that the use of NE and PE to correct hypotension in women with preeclampsia resulted in improved neonatal pH values and base excess (BE) values compared to ephedrine administration (7.32 ± 0.02 vs. 7.32 ± 0.02 vs. 7.31 ± 0.03; and 0.2 ± 1.9 vs. −0.2 ± 1.6 vs. −1.3 ± 2.9). The previous evidence has demonstrated that ephedrine can induce neonatal acidemia or acidosis by activating fetal β receptors through the placental barrier. Furthermore, the degradation of NE and PE within the placental barrier contributes to a more favorable outcome in normotensive women (Lim et al., 2018). The effects of NE and PE bolus on neonatal outcome were compared by Mohta et al. (2021), and no significant difference in umbilical artery pH was found between the two drugs (7.27 ± 0.06 vs. 7.26 ± 0.06, *p* = 0.903). In a study that focused primarily on neonatal umbilical arterial blood pH and included both bolus administration and prophylactic infusion, NE demonstrated non-inferiority to PE (Ngan Kee et al., 2020). Ikeda et al. (2023) revealed that within the context of administering prophylactic NE, the presence of low CO (defined as cumulative time >25% accompanied by a CO < 80% of baseline value) exerted a significant impact on the neonatal BE value (−2.66 ± 1.7 vs. −1.81 ± 1.6). Future investigation on neonatal outcome of preeclampsia may provide additional insights into the potential benefits of NE.

The study has some limitations. Firstly, we employed FloTrac/Vigileo for hemodynamic monitoring, which is a minimally invasive and effectively applied technique in obstetric hemodynamic monitoring (Matsota et al., 2015). Currently, non-invasive pulse wave analysis yields equally effective results. Secondly, we adopted an efficacy ratio of 12.5:1 between PE and NE to simplify the preparation and optimize the blind configuration. However, it is important to note that varying efficacy ratios may have an impact on the monitoring results of hemodynamics indicators and their maximal changes after vasopressor application. The doses of 6 µg NE and 75 µg PE administered may be considered relatively higher, and further evidence is required to determine the optimal dosage for preeclamptic women. Thirdly, a 3 - minute assessment window could theoretically favor norepinephrine due to its rapid clearance, and this may affect the maximal changes in hemodynamic indicators. Finally, focusing solely on women who experienced hypotension may limit the generalizability of our study. Conversely, incorporating all patients and performing a subgroup analysis of those who received norepinephrine or phenylephrine would provide stronger evidence regarding hemodynamic changes in preeclamptic women.

In conclusion, significant hemodynamic changes still occur in preeclamptic women with severe features after receiving spinal anesthesia, and both NE and PE can effectively rescue the hypotensive events following spinal anesthesia, exhibiting comparable efficacy. NE demonstrates superior capability in maintaining CO and HR.

## Data Availability

The original contributions presented in the study are included in the article/supplementary material, further inquiries can be directed to the corresponding author.
